# A social return on investment analysis of a free meal program to support rural cancer patients and caregivers travelling for treatment

**DOI:** 10.1007/s00520-025-09662-9

**Published:** 2025-06-20

**Authors:** Elizabeth A. Johnston, Xanthia E. Bourdaniotis, Susannah K. Ayre, Megan Oster, Deanna Romain, Belinda C. Goodwin

**Affiliations:** 1 Viertel Cancer Research Centre, Cancer Council Queensland, Fortitude Valley, QLD Australia; 2 School of Exercise and Nutrition Sciences, Queensland University of Technology, Kelvin Grove, QLD Australia; 3 Population Health Program, QIMR Berghofer Medical Research Institute, Herston, QLD Australia; 4 School of Psychology, University of Queensland, St Lucia, QLD Australia; 5 Centre for Health Research, University of Southern Queensland, Springfield, QLD Australia; 6 School of Population and Global Health, University of Melbourne, Carlton, VIC Australia

**Keywords:** Cancer survivors, Food security, Nutrition, Program evaluation, Regional and remote, Supportive care

## Abstract

**Purpose:**

Travelling for cancer treatment imposes financial burden and disrupts routines, including food access and preparation. This study assessed the social return on investment (SROI) and acceptability of providing a free, healthy meal to rural cancer patients and caregivers on arrival at a city-based accommodation lodge operated by a not-for-profit cancer support organisation.

**Methods:**

Baseline survey data were collected from lodge guests over an 8-week period, and again during an 8-week Intervention period where guests received a free meal from an on-site fresh food vending machine on the day of arrival. Descriptive statistics, qualitative analyses, and concurrent forecast and evaluative SROI analyses were conducted to evaluate the free meal program.

**Results:**

During Baseline and Intervention phases, 344 guests stayed at the lodge, with 178 (52%) and 106 (31%) surveys completed, respectively. From Baseline surveys, guests spent on average 60 min (range: 5–180 min) preparing and/or acquiring their meal on arrival, and paid AU$15.00 per person (range: AU$1.50–$50.00) for their meal and AU$10.00 per person (range: AU$3.00–$50.00) in travel/delivery costs. From Intervention surveys, guests valued the time saved preparing and/or acquiring a meal (90%), costs saved on travel/delivery (83%) and meals (80%), and a healthier or higher protein meal (60%). Every dollar invested in the meal program generated an estimated AU$3.06 of social value. For acceptability, meal enjoyment and satisfaction were higher among the Intervention group compared to Baseline (90% vs. 54%, 92% vs. 81%, respectively).

**Conclusion:**

A free meal program for rural cancer patients and caregivers travelling for treatment generated a positive SROI and was highly acceptable.

**Supplementary Information:**

The online version contains supplementary material available at 10.1007/s00520-025-09662-9.

## Introduction

For people living in rural areas, a cancer diagnosis often requires regular or extended travel to a major city for specialist treatment and follow-up. This travel disrupts everyday life for both the person diagnosed with cancer and their family members or friends (i.e., caregivers) who accompany them [1, 2]. These disruptions can include reduced participation in paid employment [3], coupled with substantial out-of-pocket costs for travel-related expenses, including transport, accommodation, and parking [2, 4]. Among rural cancer patients, higher levels of financial toxicity have been associated with poorer psychosocial health, including increased symptoms of anxiety and depression and reduced social functioning [5].

Community cancer support organisations play a key role in reducing the financial impact of cancer and its treatment for rural cancer patients and their families through free or subsidised support services [6]. For example, not-for-profit organisations in Australia [7] and the United States [8] offer subsidised accommodation at dedicated lodges within close proximity of major hospitals and treatment centres for rural cancer patients and their caregivers. For many, these lodges are ‘a home away from home’, for several days to weeks, with some staying for months while they receive complex treatments or multiple therapies.

In recent interviews with rural caregivers, many of whom had stayed at a subsidised accommodation lodge during their loved one’s cancer treatment, caregivers described how travelling to a major city for cancer treatment presents several challenges to maintaining health-promoting behaviours, including following a healthy diet [9]. These challenges included higher food costs in major cities, limited cooking facilities, and reliance on convenience foods while away from home, as well as less time available for food preparation and skipping meals due to the additional caregiving responsibilities and fatigue [9]. These findings highlighted the need for organisation-level interventions to improve both physical and economic access to healthy food for rural cancer patients and caregivers staying in subsidised accommodation lodges.

This paper reports on the social return on investment (SROI) and acceptability for providing rural cancer patients and their caregivers with a free, healthy meal on arrival at a subsidised accommodation lodge in a major city, operated by a not-for-profit cancer support organisation. Traditionally, program evaluations in healthcare analyse costs and benefits in terms of money that is spent or saved [10]. Through using an SROI approach to program evaluation, this study considers the broader value of changes experienced by recipients of the free meal program, and whether these changes were of benefit to them [11]. Acceptability was assessed alongside the SROI to identify whether the free meals provided were suitable and satisfactory for this population group. It was hypothesised that providing a free, healthy meal on arrival would generate a positive SROI for lodge guests, through reducing food preparation time and costs and improving the nutritional quality of their meal, and be highly acceptable to lodge guests.

## Methods

The MEAL project (Making it Easy to Access a meal on arrival at the Lodge) was designed as a quasi-experimental intervention study, with participant recruitment and data collection for the purpose of estimating its SROI and evaluating its acceptability as a service for improving access to a meal on arrival at the lodge [12]. All monetary data collected and reported in this study were Australian Dollars (AU$). This research was approved by the University of Southern Queensland Human Research Ethics Committee (Approval #ETH2024-0111) and conducted in accordance with the ethical standards of the 1964 Declaration of Helsinki and its later amendments.

### Social return on investment (SROI) analyses

#### Stage 1: Establish scope and identify stakeholders

The project team included researchers, Accredited Practising Dietitians, a lodge manager, and a senior supportive care consultant at Cancer Council Queensland (CCQ), a not-for-profit organisation for cancer prevention, support, and research [13]. The project was conducted at CCQ’s accommodation lodge located in Queensland’s capital city, Brisbane. Each year, the 34-room lodge provides subsidised accommodation for > 1700 cancer patients and caregivers who need to travel more than 50 km (31 miles) for cancer treatment or follow-up in Brisbane. As such most guests have travelled from a regional, rural, or remote area. Each room has a kitchenette, including a small refrigerator and microwave. A communal kitchen provides additional cooking facilities for shared use among guests.

The project included an initial 8-week Baseline phase of data collection, followed by the installation of an on-site fresh food vending machine at the lodge. During a subsequent 8-week Intervention phase of data collection, all lodge guests received a prepaid card on arrival entitling them to a free meal from the vending machine (valued at AU$12.70). An 8-week period for the Baseline and Intervention phases was selected to allow for monthly variation in guest characteristics and numbers.

Meal options available via the supplier were reviewed by dietitian team members to ensure meals provided at least 10 grams of protein, considered a ‘good source’ of protein by Food Standards Australia New Zealand [14]. The focus on protein was based on nutrition guidelines for cancer patients recommending protein-rich meals during cancer treatment [15], while balancing general healthy diet recommendations for caregivers, including lean meats, legumes, and vegetables [16]. Vegetarian meals that did not meet this minimum protein requirement were included in the vending machine to offer variety and cater for specific dietary preferences.

Meals were locally prepared and dispensed chilled via the vending machine. Most meals required reheating before consumption, except for cold meal options such as salads. The meal supplier was not involved in conducting, analysing, and reporting this study, and CCQ did not receive any financial compensation from the meal supplier.

To estimate the SROI, both forecast and evaluative analyses were undertaken. The forecast analysis, conducted after the Baseline phase and before the Intervention phase, enabled testing of data collection and analysis tools and estimated how much social value will be generated if the program meets its intended outcomes [12]. This forecast analysis also provided a basis for comparison with actual outcomes. The evaluative analysis, conducted after the Intervention phase, estimated the social value generated for lodge guests based on actual outcomes data and can be used to justify continued investment in the program [12]. By conducting both analyses, the study was able to validate the forecasted values, ensuring that the intervention’s actual impact was in line with the expected outcomes. Both the forecast and evaluative analyses were conducted using the eight Principles of Social Value and the six stages of analysis outlined in *The Guide to SROI* [12, 17]. Online Resource 1 demonstrates how the SROI principles were applied in this study.

Project scope was established from interviews with rural caregivers conducted by the first and senior authors to understand how caring for someone with cancer affects rural caregivers’ health behaviours and support seeking [9, 18]. From these findings, the project team developed a proposal for the free meal program (i.e., the MEAL project) to estimate the SROI and assess acceptability of providing lodge guests with a free, healthy meal on arrival via an on-site vending machine.

#### Stage 2: Map outcomes

**Fig. 1:**
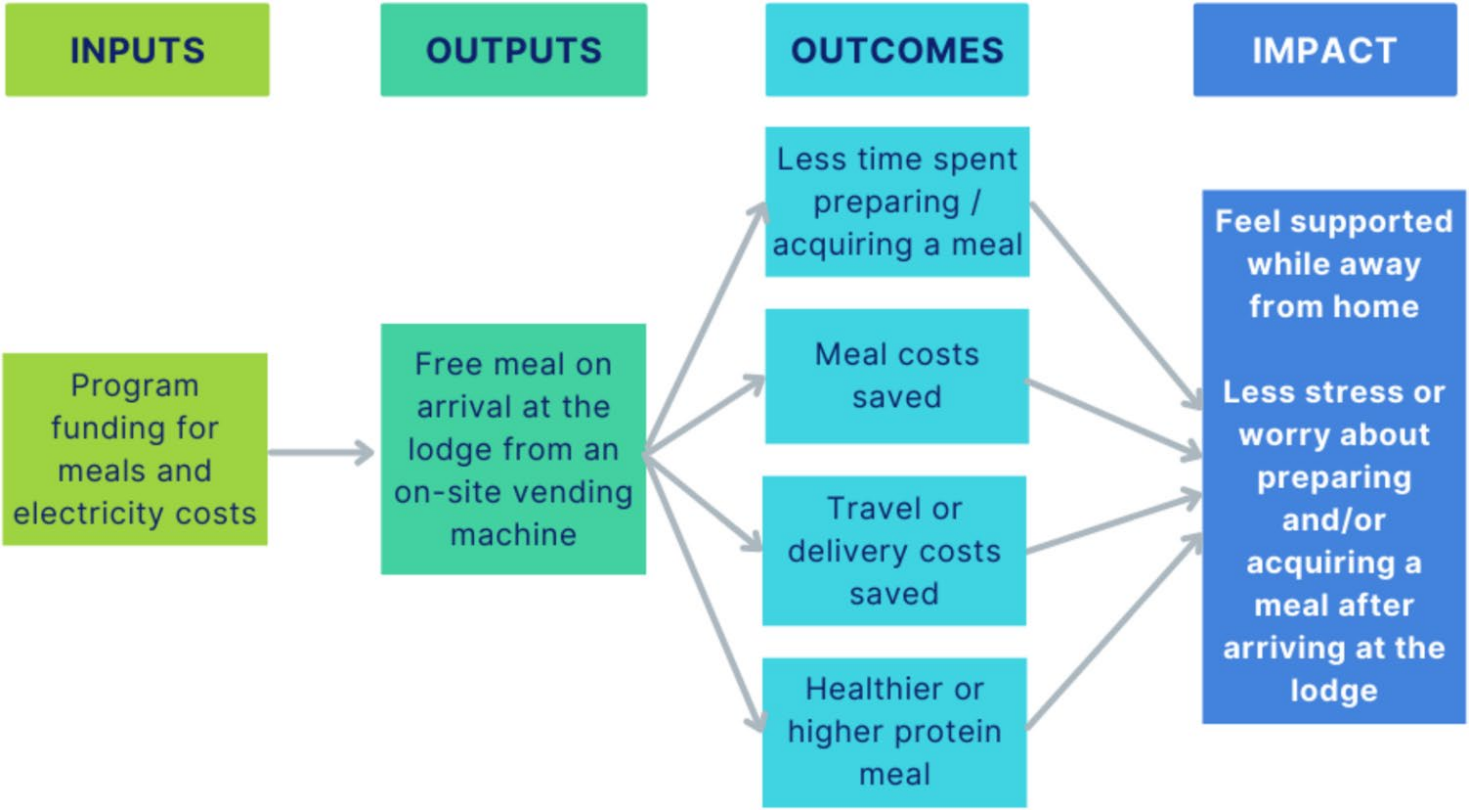
Impact Map for the free meal program included in the MEAL project showing inputs, outputs, outcomes, and hypothesised impact

The research team developed an Impact Map (or Theory of Change) to conceptualise the relationship between anticipated inputs, outputs, outcomes, and impact of the free meal program (Fig. 1). Inputs included program funding from CCQ’s Viertel Cancer Research Centre which covered the prepaid meal cards used by lodge guests and the electricity costs for running a refrigerated vending machine at the lodge. Unused meal cards were not included in inputs as CCQ was only invoiced for the meal cards that were used. No further costs were incurred by CCQ as the vending machine supplier provided free installation, on-site maintenance, and restocking of meals as per their standard business model. There was also no delivery, washing up, or cleaning fees associated with the meals. The meal supplier’s costs were not included in the SROI analyses as the aim was to evaluate the social return on CCQ’s investment in the program.

Four discrete outcomes were selected based on the barriers to accessing healthy meals while staying at the lodge reported by guests in the previous interview study [9]: (i) time saved preparing or acquiring a meal, (ii) meal costs saved, (iii) travel or delivery costs saved, and (iv) provision of a healthier or higher protein meal. All four outcomes included in the SROI analyses as they are not dependent on each other (i.e., ‘discrete’), the SROI would be misrepresented without it (i.e., ‘material’), and identified by lodge guests.

From the previous interview study [9], it was hypothesised that the free meal program would result in rural cancer patients and caregivers feeling supported while travelling for treatment. An additional impact of the free meal program was identified from qualitative feedback on Baseline surveys (less stress or worry about preparing and/or acquiring a meal after arriving at the lodge). This item was subsequently added to the Impact Map (Fig. 1) and Intervention survey. To avoid double-counting, the hypothesised impacts of the free meal program (referred to in the Impact Map as the ‘Impact’) were not included in the SROI analyses as these were the result of the four outcomes measured.

#### Stage 3: Evidence outcomes and give them a value

Two phases of data collection were conducted pre- and post- implementation of the free meal program (i.e., Baseline and Intervention surveys, respectively). Briefly, from February to April 2024, lodge staff distributed paper copies of the Baseline survey to all lodge guests via their ‘Welcome pack’ on arrival. After returning a completed survey to reception, guests received an AU$10 EFTPOS card as a thank you for their time. From June to August 2024, all guests received a prepaid meal card and a copy of the Intervention survey via their ‘Welcome pack’ on arrival. Use of the prepaid meal cards and additional meals purchased from the vending machine were monitored until the end of September 2024.

Table 1 outlines the data collected in each survey and its use in this study. For the forecast and evaluative SROI analyses, the number of people who anticipated or experienced each of outcome as a benefit (i.e., of value to them), respectively, was used to evidence each of the four outcomes.

**Table 1:**
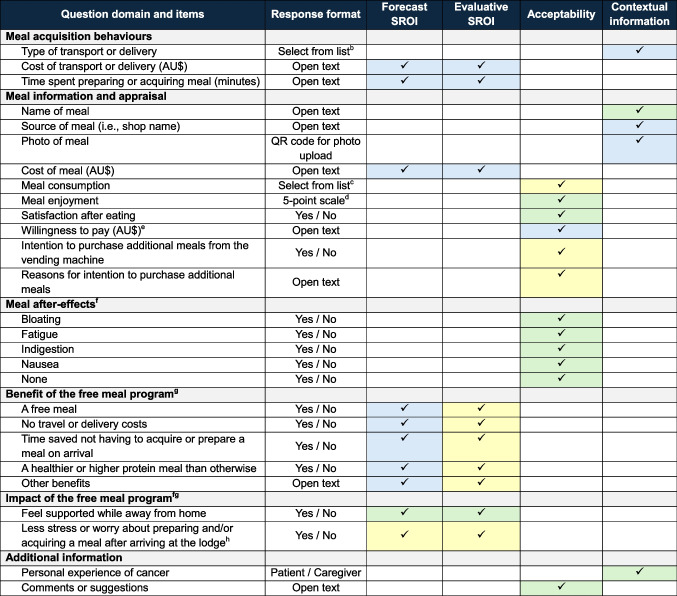
Summary of data items included in the Baseline and Intervention surveys and use in the MEAL project^a^

^a^The tick symbol (‘✓’) indicates that the survey item was used for the analysis listed in the column headings. Blue = Data from Baseline survey. Yellow = Data from Intervention survey. Green = Data from both Baseline and Intervention surveys

^b^Options included bus, taxi, Uber, private car, walking, meal delivered to lodge, meal bought on the way to lodge, meal brought from home

^c^Options included less than half, about half, all or almost all

^d^Scale ranged from 1 (did not enjoy) to 5 (very enjoyable)

^e^Participants were asked how much they would be hypothetically willing to pay if they were to receive a meal on arrival at the lodge

^f^Meal after-effects were selected based on common gastrointestinal symptoms that may occur immediately following consumption of a meal

^g^The Baseline survey collected information on anticipated benefits and impact of receiving a free meal on arrival at the lodge. The Intervention survey collected information on experienced benefits and impact of the free meal program. Benefits are conceptualised in the Impact Map as Outcomes

^h^Additional impact added to Intervention survey based on open-text responses on Baseline survey

Indicators for each outcome and financial proxies are shown in Table 2. The time-related outcome was valued using the median estimate from Baseline surveys for the number of hours that would be saved on meal preparation or acquisition, multiplied by the minimum hourly wage in Australia (AU$24.10 as of July 2024) [19]. Cost-related outcomes were valued using the median dollar values reported in Baseline surveys for meal costs and transport/delivery. For the nutrition-related outcome, the typical cost of purchasing a healthier version of a meal in Australia (e.g., sushi roll or burrito with brown rice instead of white rice) was used as a financial proxy (minimum AU$0.60 per meal). This valuation technique is known as revealed preference, where the value of the outcome is inferred from the price of related market-traded goods [12].

**Table 2:** Qualitative evidence for outcomes included in the social return on investment (SROI) analysis of the free meal program and data used for the outcome indicators and financial proxies

Outcome	Example quote from interviews with rural caregivers^a^	Indicator for outcome^b^	Financial proxy for outcome
Time saved preparing or acquiring a meal	“You spend a lot of time in hospital and by the time you leave, you think, oh, I’ll just get a burger from across the road and that’ll be my tea… it’s exhausting, draining, sitting around at hospital all the time.”	Number of people who reported saving time was/would be a benefit	Minimum hourly wage in Australia as of July 2024 (AU$24.10)^c^ multiplied by median number of hours saved on meal preparation/acquisition reported in the Baseline survey
Meal cost saved	"Living out here and then going into the city… it's overwhelming, let alone trying to find food that is affordable."	Number of people who reported that saving on meal costs was/would be a benefit	Median cost of meals reported in the Baseline survey
Travel or delivery saved	"You get to the lodge, and you can't afford to go and get a taxi around the corner to get groceries."	Number of people who reported that saving on travel/delivery costs was/would be a benefit	Median cost of travel/delivery reported in the Baseline survey
Provision of a healthier or higher protein meal	"It’s just when we’re down in [major city] – yeah, you don’t eat too well. Because you can’t get to a decent shopping centre to buy your proper meat and veggies."	Number of people who reported that having a healthier or higher protein meal than what they would have otherwise eaten was/would be a benefit	Typical cost of purchasing a healthier version of a meal (e.g., brown rice sushi or burrito instead of white rice)^d^

^a^Johnston EA, Collins KE, Vicario JN, Sibthorpe C, Ireland MJ, Goodwin BC. Changes in rural caregivers’ health behaviors while supporting someone with cancer: A qualitative study. Cancer Med. 2024;13(7):e7157

^b^The forecast SROI analysis used data collected in the Baseline survey on anticipated benefits to indicate the outcomes. The evaluative SROI analysis used data collected in the Intervention survey on benefits experienced to indicate the outcomes

^c^From Fair Work Ombudsman: https://www.fairwork.gov.au/pay-and-wages/minimum-wages

^d^Estimated using the revealed preference technique

#### Stage 4: Establish impact

The impact value for each outcome was determined by multiplying its financial value (as defined in Stage 3) by the number of people who anticipated each outcome as a benefit (for forecast SROI analysis using Baseline survey data) or who experienced each outcome as a benefit (for evaluative SROI analysis using Intervention survey data). To account for the impact caused by external factors, each outcome was assessed for deadweight, displacement, attribution, and drop-off (see definitions in Online Resource 1) [12]. As the free meal program was designed to address a gap in service delivery, and the outcomes experienced by lodge guests did not continue beyond the point of delivery, the impact value of outcomes was only adjusted for displacement, determined from Baseline survey data on meal acquisition behaviours. Total impact value (AU$) was calculated by summing the adjusted value for each outcome.

#### Stage 5: Calculate the SROI

A final ratio for both the forecast and evaluative SROI analyses was calculated by dividing the total impact value of outcomes (from Stage 4) by the value of inputs (from Stage 2), providing estimates of the social value generated for lodge guests for every dollar CCQ invested in the free meal program. To examine the effect of meal subsidy on the social value generated, two sensitivity analyses were conducted with varied levels of investment: (i) partial subsidy (AU$5.00 per meal), and (ii) no subsidy.

#### Stage 6: Report, use, and embed

The SROI analyses were shared with key stakeholders via organisation-wide presentation, posters at national oncology and dietetics presentations, and social media posts. Findings were used to seek ongoing funding to embed and expand the program across CCQ’s lodges.

### Acceptability

Acceptability was assessed by comparing meal enjoyment, satisfaction, and meal after-effects in Intervention vs Baseline surveys (Table 1). Meal after-effects were selected based on common gastrointestinal symptoms that may occur immediately following consumption of a meal [20] and aimed to capture any potentially negative outcomes of the free meal program. Additional items from the Intervention survey used to assess acceptability included meal consumption, intention to purchase additional meals from the vending machine, willingness to pay if the meal was not free, and qualitative feedback on the meals. Quantitative survey data were summarised using descriptive statistics. The free meal program was considered acceptable if 80% or more of guests who completed the Intervention survey indicated that they: (i) consumed all or almost all of their meal, (ii) scored 4 or 5 for meal enjoyment, (iii) were satisfied after eating their meal, and (iv) experienced no meal after-effects. Qualitative data were coded using content analysis [21] by one researcher (XB) then reviewed by a second researcher (EJ).

### Contextual information collected in surveys

Both the Baseline and Intervention surveys collected data to provide contextual information to the SROI and acceptability analyses (Table 1). Quantitative survey data were summarised using descriptive statistics. In the Baseline survey, the name and source of meal were used to estimate the energy and protein content of meals prior to the free meal program using nutrition information available online via grocery stores, restaurants, fast food chains, and Easy Diet Diary [22]. Energy and protein content of meals reported in Intervention surveys were extracted from nutrition information available online from the meal supplier and verified via email [23]. For each meal reported in Baseline and Intervention surveys, the proportion of energy intake from protein was calculated by multiplying grams of protein by its Atwater factor (16.7 kilojoules), divided by kilojoules per serve (reported as a percentage).

## Results

### Meal acquisition behaviours prior to the free meal program

During the 8-week Baseline phase prior to the free meal program, surveys were completed by 178 (52%) of 344 lodge guests, including 105 cancer patients and 64 caregivers (*n* = 9 unknown). As shown in Online Resource 2, most guests (62%) purchased their first meal after arrival, with 23% bringing a meal from home, and 14% purchasing their meal during transit to the lodge. One guest received a free meal on arrival from another lodge guest. Of the 134 guests who purchased their meal during transit to the lodge or after arrival, meals were mostly sourced from casual dining establishments (35%), grocery stores (26%), fast food chains (18%), and nearby hospitals (14%). For those who purchased their meal after arrival (*n* = 109), meals were obtained via car (37%), Uber (13%), and taxi (13%) or delivered to the lodge (19%).

### Uptake of the free meal program

During the 8-week Intervention phase, 222 (65%) of 344 lodge guests used their prepaid meal card, with an additional 88 meals purchased. The most popular meals selected from the vending machine were lasagna (21%), slow cooked lamb with vegetables (21%), beef brisket with vegetables (13%), spaghetti Bolognese (10%), green chicken curry (9%), and butter chicken (9%). Almost half of the guests who used their prepaid meal card completed an Intervention survey (*n* = 106; 48%), including 62 cancer patients and 36 caregivers (*n* = 8 unknown).

### Outcomes of the free meal program

Of the guests who completed an Intervention survey as part of the free meal program, 90% reported benefiting from the time saved preparing or acquiring a meal. From data collected in the Baseline surveys prior to the free meal program, guests estimated spending 60 min on average (range: 5–180 min) preparing or acquiring their first meal on arrival.

Of the guests who completed an Intervention survey as part of the free meal program, 83% and 80% reported that no travel or delivery costs and no meal costs was a benefit to them, respectively. From data collected in the Baseline surveys, travel or delivery cost for guests who purchased their meal after arrival was AU$10.00 per person on average (range: AU$3.00–$50.00). On average, guests paid AU$15.00 per person (range: AU$1.50–$50.00) for their first meal on arrival at the lodge.

Meals consumed in the Intervention phase had on average, a higher amount of protein (I: 26 g vs. B: 23 g) and higher proportion of energy from protein (I: 24% vs. B:18%), compared to meals in the Baseline phase (Table 3 and Online Resource 3), with 60% of guests in the Intervention phase reporting access to a healthier or higher protein meal than what they would have otherwise been able to prepare or buy after arriving at the lodge was a benefit to them.

**Table 3:** Acceptability, outcomes, and impact of the free meal program based on comparison of survey data collected in the Baseline and Intervention phases of the MEAL project

	Baseline (*N* = 178)	Intervention (*N *= 106)
Acceptability^a^		
Meal enjoyment, N (%)		
Did not enjoy	22 (13)	5 (5)
Neutral	57 (33)	5 (5)
Enjoyable	91 (54)	87 (90)
Did not complete	8	9
Satisfied after eating meal, N (%)		
Yes	136 (81)	90 (92)
No	32 (19)	8 (8)
Did not complete	10	8
After-effects of meal, N (%)		
At least one	40 (24)	6 (6)
None	124 (76)	95 (94)
Not reported	14	5
Types of after-effects of meal, N (%)^b^		
Fatigue	18 (45)	2 (33)
Bloating	14 (35)	0 (0)
Indigestion	10 (25)	3 (50)
Nausea	4 (10)	3 (50)
Meal consumption, N (%)		
Less than half	-	1 (1)
About half	-	8 (8)
Almost all or all	-	90 (91)
Not reported	-	7
Intent to purchase additional meals from machine at own expense, N (%)		
Yes	-	67 (70%)
No	-	29 (30%)
Not reported	-	10
Outcomes used in SROI analyses		
Anticipated or experienced benefits, N (%)^c^		
Save time preparing or acquiring a meal	148 (88)	94 (90)
Free meal	136 (80)	84 (80)
No travel or delivery costs	127 (75)	87 (83)
Healthier or higher protein meal	126 (75)	63 (60)
Did not complete	9	1
Nutritional quality of meal		
Energy (kJ), median (range)	1972 (167–6830)	1310 (1130–2530)
Protein (g), median (range)	23 (1–59)	26 (6–36)
% of total energy from protein, median (range)	18 (3–51)	24 (9–38)
Impact		
Feel supported while away from home, N (%)	135 (80)	92 (88)
Less stress or worry about preparing and/or acquiring a meal after arriving at the lodge, N (%)^d^	-	97 (92)
Did not complete	9	1

^a^Baseline survey collected information on acceptability of first meal prepared/acquired on arrival by lodge guests. Intervention survey collected information on acceptability of free meal provided on arrival

^b^Multiple after-effects could be selected by guests. Percentages were determined based on the total number of participants who reported at least one after-meal effect (*n* = 40 for baseline, *n* = 6 for intervention)

^c^Benefits are conceptualised in the Impact Map as Outcomes. Benefits of the free meal program as anticipated by the Baseline group, and as experienced by the Intervention group

^d^Additional impact added to Intervention survey following qualitative feedback on Baseline survey

As shown in Table 4, qualitative feedback from lodge guests who completed the open-text questions in the Baseline and Intervention surveys supported the outcomes selected for inclusion in the SROI analyses. For guests in the Intervention phase, 88% reported feeling supported while away from home and 92% reported less stress or worry about finding a meal after arriving at the lodge (Table 3).

**Table 4:** Qualitative feedback on the free meal program from lodge guests who completed the open-ended survey questions in the Baseline and Intervention phases of the MEAL project

	Baseline phase	Intervention phase
Outcomes valued in the SROI analyses		
Save time preparing and/or acquiring a meal	“I don't know where the shops are, so [the free meal service] means less driving and more time.” “Having one meal would allow time to either order [groceries] online or get your bearings.”	“Having food available 24/7 helps for early mornings or late nights. Very convenient.” “[The free meal service] made eating easy and less of a chore after long days at the hospital.”
Save money preparing and/or acquiring a meal	“A free meal would help… On other arrivals, I have purchased meals from [hospital] (milk cost over $4 per litre) until I could find public transport to shops and carry myself when unwell.” “Money is in short supply when fighting cancer.”	“I have previously used [delivery service] to get meals delivered on overnight stays at a cost of [AU]$60. I will use this service instead in future.”
Healthier or higher protein meal	“A healthy option would be better than takeaway.” “Healthier choice.”	“Nutritious and delicious protein and veg.” “Had plenty of flavour, good size portion, good quality, quick and easy to heat.” “The lamb was not too fatty, the flavours were very good.”
Anticipated impact of the free meal program		
Feel supported while away from home	“[The free meal service] would be a nice welcome gesture while we settle in.” “Providing a free meal is a great way of making sure people are eating a nice meal for their first night. It would feel like home with a cooked meal.”	“Felt very privileged and cared for so far away from home. Thank you for this wonderful service.” “It was very comforting after arriving at the lodge late, with no transport and no idea where I am. A free meal felt like some love. Thank you very much. I had two in the end.”
Additional impact of the free meal program^a^		
Less stress or worry about preparing and/or acquiring a meal after arriving at the lodge	“Would be amazing and a relief to not worry about food on arrival.” “We went to lunch as a special treat but after we spent an hour navigating stressful traffic trying to find a grocery store to get into the parking etc. So stressful.” “[It would help] not having to worry about looking for somewhere to get food or something to eat.” “It would make the end of the trip less stressful.”	“[The free meal service] relieves some stress knowing a meal is available. It's also a nice treat, being able to have a choice.” “Stress free meal when away from home. Already stressed just starting this cancer journey.” “Loved being able to get a meal here and not have to worry about organising [delivery service].”

^a^Additional impact reported in Baseline survey and subsequently added to the Intervention survey

### Social value generated through the free meal program and SROI ratio

The evaluative SROI analysis is shown in Online Resource 4. The total value of inputs was AU$1444.20 (106 meals at AU$12.70 each + $98 electricity costs). The total value of outcomes was AU$4426.14. Time saved was valued at AU$2265.40, contributing to 51% of the social value generated (reported by *n* = 94/106, saving 60 min per person on average, valued at AU$24.10 per hour). Meal cost saved was valued at AU$1260.00, contributing to 28% of the social value generated (reported by *n* = 84/106, valued at AU$15.00 per person). After adjusting for deadweight to account for the one guest in the Baseline phase who received a free meal from another guest, meal cost saved was valued at AU$1252.94. Travel or delivery cost saved was valued at AU$870.00, contributing to 20% of the social value generated (reported by *n* = 87/106, valued at AU$10.00 per person). Improved nutritional quality of meal was valued at $37.80, contributing to 1% of the social value generated (reported by *n* = 63/106, valued at $0.60 per person). No adjustments were made for displacement, attribution, or drop-off. The program did not displace other activities at the lodge, no other programs or initiatives contributed to the change that occurred, and the benefit of the program does not continue beyond receipt of a free meal (i.e., no drop-off).

When comparing the value of inputs with the social value generated, for every dollar invested in the free meal program, an estimated AU$3.06 of social value was generated for lodge guests (SROI ratio of 1:3.06). This evaluative SROI ratio is similar to the forecast SROI ratio of 1:2.94 (Online Resource 5).

### Sensitivity analysis for evaluative SROI ratio

When the meal cost (AU$12.70) was shifted to guests (i.e., no meal subsidy), the SROI ratio reduced to 1:2.33, with meal cost saved accounting for 6% of the social value generated, and the value of time saved and travel or delivery cost saved increasing to 67% and 26%, respectively (Online Resource 6). When meal cost was set at AU$7.70 (i.e., subsidised by AU$5.00), the SROI ratio reduced to 1:2.62, with meal cost savings accounting for 16% of the social value generated, and time saved and travel or delivery cost saved accounting for 60% and 23%, respectively (Online Resource 7).

### Acceptability of the free meal program

Most guests (91%) who received a free meal reported consuming all or almost all their meal. Compared to the Baseline (B) phase, both meal enjoyment and satisfaction were higher in the Intervention (I) phase (I: 90% vs. B: 54% and I: 92% vs B: 81%, respectively) (Table 3). Few guests reported negative after-effects of their free meal compared to the Baseline phase (I: 96% vs. B: 77%). Seventy percent indicated they planned to purchase additional meals from the vending machine during their stay. From qualitative feedback on Intervention surveys, those who did not plan to purchase additional meals commonly indicated they were short-stay guests (i.e., 1 night) or had brought other food with them. When asked how much they would be willing to pay for a meal on arrival at the lodge if it was *not* free, average willingness to pay was AU$15.00 (range: AU$5.00–$50.00).

## Discussion

Providing a free, healthy meal on arrival at the lodge via an on-site fresh food vending machine was highly acceptable to guests and generated a positive social return on investment (SROI). Most of the social value generated was through time saved in meal preparation, followed by reduced meal cost. While the SROI was sensitive to meal cost, it remained positive at various levels of subsidy (full, partial, none). We also observed that the relative value of the financial proxies ascribed to each outcome reflected the proportion of guests who identified each outcome as a benefit (i.e., outcomes with a higher value in the SROI analyses were reported as benefits by a higher number of lodge guests). Thus, the methods used to value the outcomes in the SROI analyses appears to align with guests’ appraisal of their relative value. Almost all guests who reported experiencing at least one benefit from the free meal program also indicated that they felt supported while away from home, the hypothesised impact of the program. Less stress or worry about acquiring a meal on arrival was an additional positive impact of the program, reported by nearly all survey respondents who received a free meal. Finally, the free meal program met a priori criteria for acceptability, including high levels of meal consumption, enjoyment, and satisfaction, and minimal after-effects.

While the need for practical assistance and financial support for people travelling for cancer treatment and follow-up is well-recognised [2, 3, 24], no similar interventions have been published. Several trials have shown that longer-term interventions, involving food pantries, food vouchers, and/or home delivery of meals or groceries have improved outcomes among cancer patients in the United States [25–27] and Denmark [28], including better food security, diet quality, physical capability, and quality of life. Findings from studies among people with heart disease, diabetes mellitus, and chronic kidney disease also suggest that providing longer-term (~ 12 weeks) access to medically tailored meals can significantly improve adherence to dietary recommendations for chronic disease control, with improvements in body weight, blood pressure, and lipid profile [29]. In our study, the high turnover of short-stay guests meant assessing the value of long-term, on-site access to healthy meals at the lodge was beyond scope. However, guests were able to purchase additional meals throughout their stay for approximately half the average meal cost and less than the median ‘willingness to pay’ reported by guests pre-intervention.

Based on our findings, the main benefit of the free meal program was the practical and financial support provided. Studies evaluating the effectiveness of other short-term practical or financial support programs have focused on transportation and accommodation services for patients with chronic conditions [30, 31]. For example, a systematic review of interventions for non-emergency medical transportation found that taxi or bus vouchers and free ride-sharing services were associated with fewer missed healthcare appointments; however, there was insufficient evidence of their association with other healthcare utilisation and cost outcomes [31]. Another systematic review examining the effectiveness of the patient hotel model, which combines non-acute hospital care with hospitality, found patients reported increased independence, privacy, and comfort, which contributed to improved well-being [30]. Thus, incorporating a free meal program into existing accommodation services could provide a more comprehensive and valuable support service that meets the basic physiological needs of people who must relocate during cancer treatment [32].

### Strengths and limitations

This study used the SROI framework, a recognised method for program evaluation in public health [33]. Unlike other approaches that rely on standardised health or economic outcomes, such as a cost-effectiveness analysis, SROI captures only the benefits that stakeholders identify as meaningful. This strengthens the analysis by ensuring that outcomes are not assumed to be universally beneficial but rather reflect the values and experiences of program participants. The use of SROI to evaluate cancer supportive care interventions is relatively novel with only two studies completed [34, 35] and one underway [36]. In this study, pre-post data collection enabled robust estimates for financial proxies of outcomes [10]. While sample size is not a key principle of SROI, and the focus is on using the best available data to account for stakeholders’ experiences of change and the value ascribed to these changes, this analysis included perspectives from more than 280 rural cancer patients and caregivers. The surveys did not collect demographic information, so it is unknown how respondents differed from non-respondents, and between Baseline and Intervention phases.

By conducting a forecast analysis pre-intervention, we could verify the evaluative SROI ratio calculated post-intervention, demonstrating that regardless of whether guests received a free, healthy meal on arrival or anticipated the benefits of this intervention, the value generated by the free meal program for lodge guests is triple the original investment. While shifting some or all of the meal cost to guests reduced the social value generated, the SROI ratio remained > 2 (i.e., the social value generated is more than double the value of the original investment). This finding suggests that the program continues to generate social value even with reduced financial investment, highlighting its potential for sustainability and scalability in different funding contexts. It also underscores the robustness of the positive social value generated for guests, primarily through saving time and costs associated with meal delivery or transport – benefits that are particularly important for rural cancer patients and caregivers, who often face significant challenges related to travel, including time and financial burdens [37].

Additionally, there may be broader societal costs and benefits beyond the scope of this analysis. For example, costs to the meal producer for preparing, cooking, and packaging the meals and costs to the supplier for meal delivery and administration. Having a leading not-for-profit cancer support organisation as a client may provide reputational benefits for both the meal producer and supplier. Further, the additional meals purchased by guests were not within scope of the SROI analyses for the free meal program but could provide additional social value beyond the immediate intervention. Additionally, reasons for guests not using their free meal card in the Intervention phase were unknown as surveys were only completed by those who did participate in the free meal program. Finally, if using an SROI approach to evaluate longer-term meal provision at the lodge, additional adjustments may be needed (e.g., displacement, attribution, drop-off) [12], although there may be economies of scale, such as discounted meals from the supplier. Meal costs may also be subject to inflation over time, although the value of outcomes may increase concurrently.

## Conclusions

For rural cancer patients and caregivers staying at a city-based subsidised accommodation lodge during cancer treatment or follow-up, receiving a free, healthy meal on arrival via an on-site fresh food vending machine was highly acceptable and beneficial. Through the time and money saved, and provision of a meal with higher nutritional quality, this initiative generated a positive social return on investment for lodge guests estimated at triple the value of the money required to provide the meals. This study provides evidence for a practical intervention to support rural cancer patients and their families travelling for treatment and demonstrates the use of the social return on investment framework for evaluating a supportive care intervention in the cancer setting.

## Supplementary Information

Below is the link to the electronic supplementary material.

Supplementary file1 Online Resource 1. Application of the eight social return on investment (SROI) principles in the MEAL project, Online Resource 2. Meal acquisition behaviours and costs reported in surveys during the Baseline phase of the MEAL project (N=178), Online Resource 3. Photos of meals prepared or acquired on arrival submitted by lodge guests who completed a Baseline survey as part of the MEAL project (DOCX 1440 KB)

Supplementary file2 Online Resource 4. Evaluative Social Return On Investment (SROI) analysis of the MEAL project with full subsidy for meal costs, Online Resource 5. Forecast Social Return On Investment (SROI) analysis of the MEAL project with full subsidy for meal costs, Online Resource 6. Evaluative Social Return On Investment (SROI) analysis of the MEAL project with no subsidy provided for meal costs, Online Resource 7. Evaluative Social Return On Investment (SROI) analysis of the MEAL project with partial subsidy provided for meal costs (XLSX 54.2 KB)

## Data Availability

The data that support the findings of this study are available on request from the corresponding author. The data are not publicly available due to privacy or ethical restrictions.
