# Supplementary material for: A social return on investment analysis of a free meal program to support rural cancer patients and caregivers travelling for treatment
Source: Support Care Cancer. 2025 Jun 20;33(7):602. doi: 10.1007/s00520-025-09662-9 (PMC12181214; doi:10.1007/s00520-025-09662-9)
Supplement: Supplementary file 1 — Supplementary file1 Online Resource 1. Application of the eight social return on investment (SROI) principles in the MEAL project, Online Resource 2. Meal acquisition behaviours and costs reported in surveys during the Baseline phase of the MEAL project (N=178), Online Resource 3. Photos of meals prepared or acquired on arrival submitted by lodge guests who completed a Baseline survey as part of the MEAL project (DOCX 1440 KB) [file 520_2025_9662_MOESM1_ESM.docx]

**Online Resource 1.** Application of the eight social return on investment (SROI) principles in the MEAL project

| **SROI Principle and Definition^a^** | **Application in MEAL project** |
| --- | --- |
| 1. **Involve stakeholders** | - The need to address barriers to accessing healthy meals while travelling for cancer treatment was identified from interviews with rural caregivers about the impact of caring for someone with cancer on their health and wellbeing. - Rural caregivers’ experiences of accessing food on arrival at CCQ’s lodges were used to design the MEAL project and identify outcomes to measure and how they are valued. - A project group was assembled including CCQ executive and senior management, dietitians, researchers, a lodge manager, and a cancer support advisor. - First author completed the ‘Social Value & SROI Training’ course hosted by Social Ventures Australia. |
| *Stakeholders are defined as the people or organisations who will experience change as a result of the project. Stakeholders are identified and involved in determining what outcomes are measured and how they are valued.* |  |
| 1. **Understand what changes** | - The MEAL project includes two phases of primary data collection with lodge guests (Baseline and Intervention surveys) to understand their anticipated and actual experiences of change from the free meal program. Both surveys capture positive and intended changes with ‘other’ response items and open-text questions to capture changes that are negative or unintended. |
| *Gather evidence to understand how change occurs, including positive and negative changes, and intended and unintended changes.* |  |
| 1. **Value the things that matter** | - Outcomes valued in the SROI analyses were identified from interviews with rural caregivers. - The Baseline and Intervention surveys collected information on anticipated and experienced benefits (i.e., outcomes), respectively. This enabled the forecast and evaluative SROI analyses to account for the number of lodge guests who anticipated or experienced each outcome as a benefit (i.e., of value to them), respectively, rather than assuming that all guests benefited from each outcome. |
| *Outcomes that are valued in the analysis were identified by stakeholders as important.* |  |
| 1. **Only include what is material** | - Decision-making for the SROI analyses is documented in the peer-reviewed paper and supplementary materials. - The spreadsheet used to conduct the SROI analyses is available in supplementary materials. - Additional survey findings that are relevant to the uptake, acceptability, and benefit of the free meal program, but were not material to the SROI analyses, are reported in the peer-reviewed paper (e.g., types of meals consumed). |
| *Ensure all relevant information and evidence is included to provide a true description of the impact.* |  |
| 1. **Do not overclaim** | - Outcomes measured in the MEAL project are discrete (i.e., no double counting). - SROI analyses considered deadweight, displacement, attribution, and drop-off.   - *Deadweight*: proportion of the outcome that would have happened anyway without the free meal program in place).   - *Displacement*: proportion of the outcome that has displaced similar outcomes from other programs resulting in less impact elsewhere.   - *Attribution*: proportion of the outcome attributed to other organisations or people.   - *Drop-off*: proportion of the outcome that will not be present in future years. - The free meal program was designed to address an unmet need and did not duplicate or overlap with other support available to lodge guests. |
| *Only include the outcomes that the project generated by accounting for other reasons that the outcome may have occurred.* |  |
| 1. **Be transparent** | - Decision-making for the SROI analyses is documented in the peer-reviewed paper and supplementary materials. - The survey findings and SROI analyses will be available online via an open-access publication. - The survey findings and SROI analyses have been shared with organisational executive and senior management to advocate for continued funding and support for lodge guests. - Social media posts will communicate findings to CCQ’s clients and networks. |
| *Demonstrate measures used to ensure accuracy and rigor, including reporting of findings to stakeholders.* |  |
| 1. **Verify the result** | - The survey findings and SROI analyses were reviewed by the project team prior to peer review for publication. - Peer review of the survey findings and SROI analyses by academic reviewers as part of publication process. |
| *Seek independent review of the analysis (level of verification needed is proportionate to the investment decisions that the analysis will inform).* |  |
| 1. **Be responsive** | - Survey findings and SROI analyses have been shared with organisational executive and senior management to advocate for continued funding and support for lodge guests. - Based on findings, the project team is investigating options for expanding the free meal program to CCQ’s other lodges and including high-protein snacks, drinks, and breakfast items. |
| *Share findings with stakeholders and use to inform future decisions.* |  |

^a^ SROI principles and definitions based on the Standards and Guidance published by Social Value UK (<https://socialvalueuk.org/standards-and-guidance/>)

**Online Resource 2.** Meal acquisition behaviours and costs reported in surveys during the Baseline phase of the MEAL project (N=178)

| **Meal acquisition behaviours** | | **N (%)** | |
| --- | --- | --- | --- |
| Timing of meal acquisition | |  | |
| Brought from home | | 41 (23) | |
| Purchased in transit to the lodge | | 25 (14) | |
| Purchased after arrival at the lodge | | 109 (62) | |
| Received from another lodge guest | | 1 (1) | |
| Not completed | | 2 | |
| Source of meals^a^ | |  | |
| Casual dining establishment | | 46 (35) | |
| Grocery store | | 34 (26) | |
| Fast food chain | | 23 (18) | |
| Nearby hospital | | 19 (14) | |
| Convenience store | | 5 (4) | |
| Fine dining restaurant | | 3 (2) | |
| Airport | | 1 (1) | |
| Not completed | | 3 | |
| Modes of travel or delivery used to acquire meal^b^ | |  | |
| Car | | 40 (37) | |
| Delivery to the lodge | | 21 (19) | |
| Uber | | 14 (13) | |
| Taxi | | 14 (13) | |
| Walking | | 12 (11) | |
| Bus | | 11 (10) | |
| **Costs** | | **Median (range)** | |
| Time spent preparing or acquiring meal on arrival at the lodge (minutes) | | 60 (5-180) | |
| Travel or delivery costs ($)**^c^** | | 10 (3-50) | |
| Meal or ingredient costs ($)**^c^** | | 15 (1.5-50) | |
| Willingness to pay for a healthy meal on arrival at the lodge (if not provided for free) ($)**^c^** | | 15 (5-50) | |

^a^ For the 134 guests who purchased their meal in transit or after arrival at the lodge.
^b^ For the 109 guests who purchased their meal after arrival at the lodge. Sums to 112 as 3 guests used 2 modes of transport to acquire their meal.
^c^ Costs are reported in 2024 Australian Dollars (AU$).

**
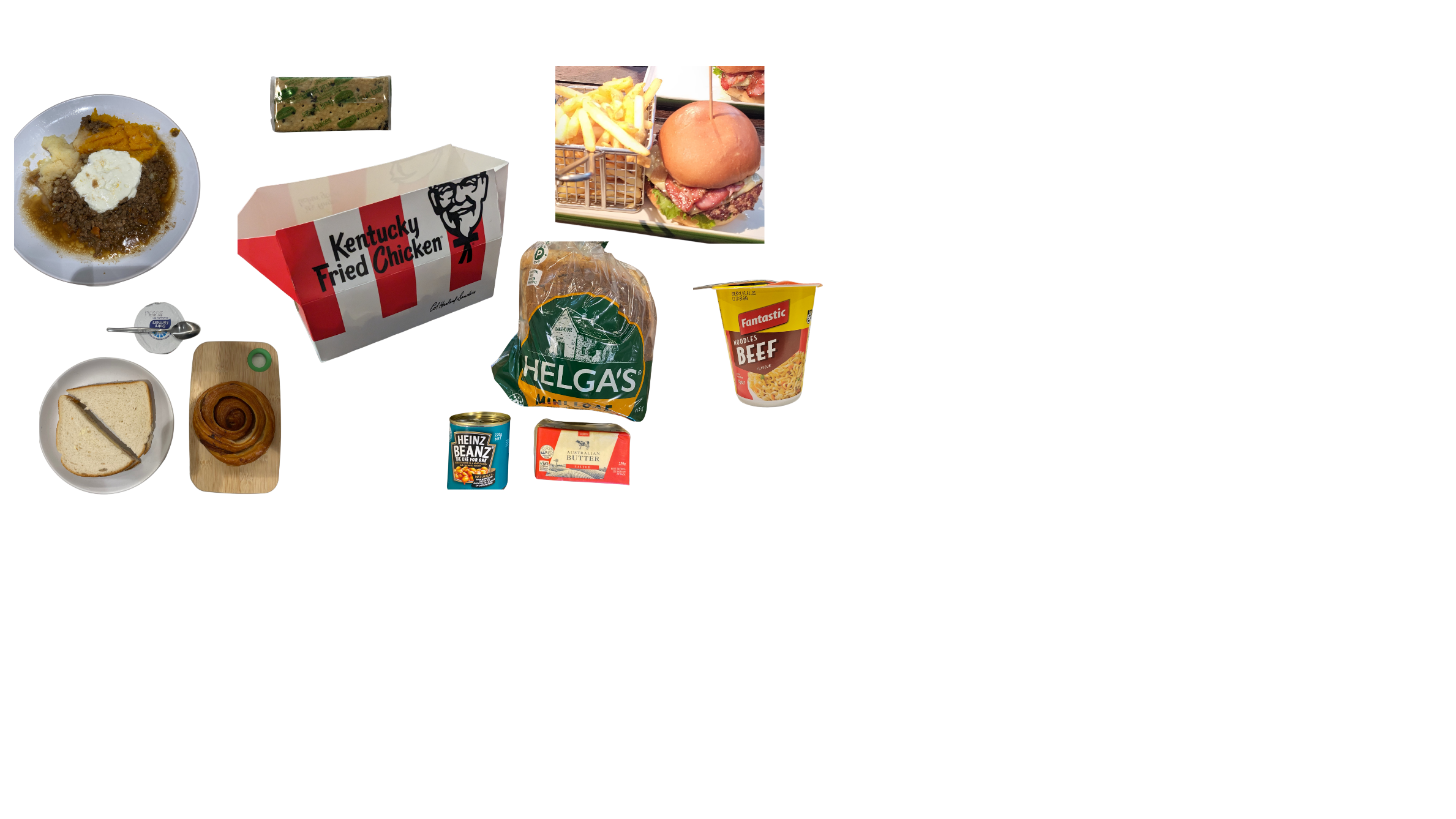
Online Resource 3.** Photos of meals prepared or acquired on arrival submitted by lodge guests who completed a Baseline survey as part of the MEAL project
